# A comprehensive map of the bovine mobilome and their epigenetic regulation of mastitis

**DOI:** 10.1007/s10142-026-01970-5

**Published:** 2026-09-16

**Authors:** Naisu Yang, Mengqi Wang, Sarah E. Abanda Mbili, Antony T. Vincent, Chengyi Song, Eveline M. Ibeagha-Awemu

**Affiliations:** 1 Département des sciences animales, Université Laval, Québec, Québec Canada; 2 Department of Animal Science and Technology, Yangzhou University, Yangzhou, Jiangsu China; 3 Sherbrooke Research and Development Centre, Agriculture and Agri-Food Canada, Sherbrooke, Québec Canada

**Keywords:** Retrotransposon, Short Interspersed Nuclear Elements (SINEs), Long Interspersed Nuclear Elements (LINEs), Endogenous retroviruses (ERVs), DNA methylation, Subclinical mastitis, Cattle

## Abstract

Retrotransposons are major components of mammalian genomes, yet their genome-wide annotation and epigenetic regulation in cattle remain incompletely characterized. Here, we present a comprehensive annotation and integrative epigenomic analysis of major retrotransposon classes in the bovine genome, with emphasis on DNA methylation patterns and their potential roles in subclinical mastitis. Using a multi-step *de novo* pipeline, we identified and classified long interspersed nuclear elements (LINEs), short interspersed nuclear elements (SINEs), and endogenous retroviruses (ERVs) in the bovine reference genome, including three LINE families, seven SINE families, and 20 ERV families. Retrotransposons accounted for ~ 40% of the genome, with LINEs representing the largest fraction. Evolutionary analysis suggested that BosL1A1, BosSINEL, BosERV1, and BosERV16 are the most recently active families. DNA methylation profiles in milk somatic cells showed consistently high levels across retrotransposons, with widespread hypermethylation in cows with *Staphylococcus aureus*–induced subclinical mastitis. We identified 20,839 differentially methylated retrotransposons, 3,306 of which overlapped differentially expressed genes. Promoter- and exon-overlapping elements showed inverse correlations between methylation and gene expression, and enriched genes are involved in immune and transport pathways. These results provide a comprehensive bovine mobilome resource and indicate that retrotransposon methylation is associated with gene regulation and host responses to mastitis.

**Supplementary Information:**

The online version contains supplementary material available at https://doi.org/10.1007/s10142-026-01970-5.

## Introduction

The mobilome, comprising all mobile genetic elements in a genome, plays a fundamental role in shaping genome structure and function. Among its major components are transposable elements (TEs), DNA sequences capable of moving or copying and replicating themselves in new genomic locations. TEs are broadly categorized into Class I (retrotransposons), which transpose via an RNA intermediate, and Class II (DNA transposons), which move through a cut-and-paste mechanism (Bourque et al. 2018). In mammalian genomes, retrotransposons are by far the most abundant and active group, representing a significant fraction of total genomic content. Retrotransposons are further divided into three major classes: Long Interspersed Nuclear Elements (LINEs), which are autonomous and encode proteins necessary for retrotransposition; Short Interspersed Nuclear Elements (SINEs), which are non-autonomous and rely on LINE machinery; and Long Terminal Repeat (LTR) retrotransposons, including endogenous retroviruses (ERVs), which are remnants of ancient viral infections (Mita and Boeke 2016). These TEs are not only numerous but have been shown to influence gene regulation, genome stability, and epigenetic programming (Cordaux and Batzer 2009; Elbarbary et al. 2016; Mita and Boeke 2016).

Retrotransposons are not merely passive genomic passengers; they serve as potent evolutionary and regulatory forces in mammalian genomes. By inserting into or near genes, retrotransposons can create structural variation, modify gene expression, and even give rise to novel regulatory elements such as alternative promoters and enhancers (Bourque et al. 2018). They also contribute to epigenetic remodeling through DNA methylation and histone modification patterns that silence or modulate their activity, with downstream effects on neighboring genes (Jansz 2019). A growing body of evidence demonstrates that many transposable element–derived sequences have been co-opted as regulatory modules in immune and stress-response pathways, often in a lineage-specific manner (Chuong et al. 2013, 2016). Notably, comparative multi-species analyses have revealed that transposable elements contribute both ancient and lineage-restricted binding sites for NF-κB and other inflammatory transcription factors, thereby shaping species-specific immune responses (Wang et al. 2025). In livestock species, retrotransposons are increasingly recognized as contributors to agriculturally and physiologically relevant traits. In cattle, Bov-A2 SINE elements have been shown to function as inducible enhancers, introducing regulatory variability across individuals and breeds and influencing transcriptional networks involved in immunity and host defense (Kelly et al. 2022). A transcriptomic study of bovine mastitis further suggested that pathogen challenge can uncover transposable element–derived regulatory elements that are dynamically engaged during inflammatory responses, highlighting their potential role in disease susceptibility and resilience (An et al. 2025). DNA methylation serves as a primary host defense mechanism against retrotransposon activation, and its perturbation has been linked to inflammatory diseases and immune dysfunction (Deniz et al. 2019; Jansz 2019).

Given the significant roles retrotransposons may play in shaping bovine genome function and phenotypic variation, it is essential to develop an accurate and comprehensive map of these elements in the cattle genome. Previous studies have shown that transposable elements comprise nearly half of the cattle genome, with LINEs and SINEs representing the most abundant classes, particularly BovB-derived elements (Adelson et al. 2009; Zimin et al. 2009). Despite these advances, most existing annotations rely heavily on repeat libraries such as Repbase and Dfam, which are biased towards model organisms. As a result, younger, cattle-specific, or recently active retrotransposons are often underrepresented or missed entirely. In addition, current catalogs rarely integrate epigenetic information or population-level variation, limiting our understanding of the regulatory and functional roles of retrotransposons. These gaps highlight the need for a comprehensive, high-resolution annotation of bovine retrotransposons that captures their structural diversity, evolutionary dynamics, and epigenetic regulation. Early genome-wide annotations of repetitive elements in cattle, most notably by Adelson et al. (Adelson et al. 2009), were based on the Btau4.0 assembly, which was limited by low contiguity and incomplete annotation. These constraints restricted the detection of recently active, lineage-specific retrotransposons and precluded detailed assessment of their functional relevance.

In this study, we leverage the latest bovine reference genome, ARS-UCD2.0, which provides substantially improved assembly continuity and gene annotation accuracy, enabling more precise identification and classification of retrotransposon families. Using this updated reference, we present a comprehensive, high-resolution annotation of bovine retrotransposons that integrates *de novo* discovery, subfamily classification, insertion age estimation, and genomic context analysis. We also genotyped selected young SINE insertions across Holstein populations to assess insertion polymorphism and distribution. Beyond sequence-level characterization, we investigated epigenetic regulation of retrotransposons by analyzing whole-genome bisulfite sequencing data from milk somatic cells of healthy cows and cows with *Staphylococcus aureus*–induced subclinical mastitis. We characterized DNA methylation patterns across retrotransposon bodies and flanking regions and identified condition-specific differentially methylated elements. Together, these analyses provide an integrated genomic, epigenomic, and population-level framework for understanding the functional and evolutionary roles of retrotransposons in the bovine genome.

## Materials and methods

### Identification of retrotransposon in the bovine reference genome

**Fig. 1:**
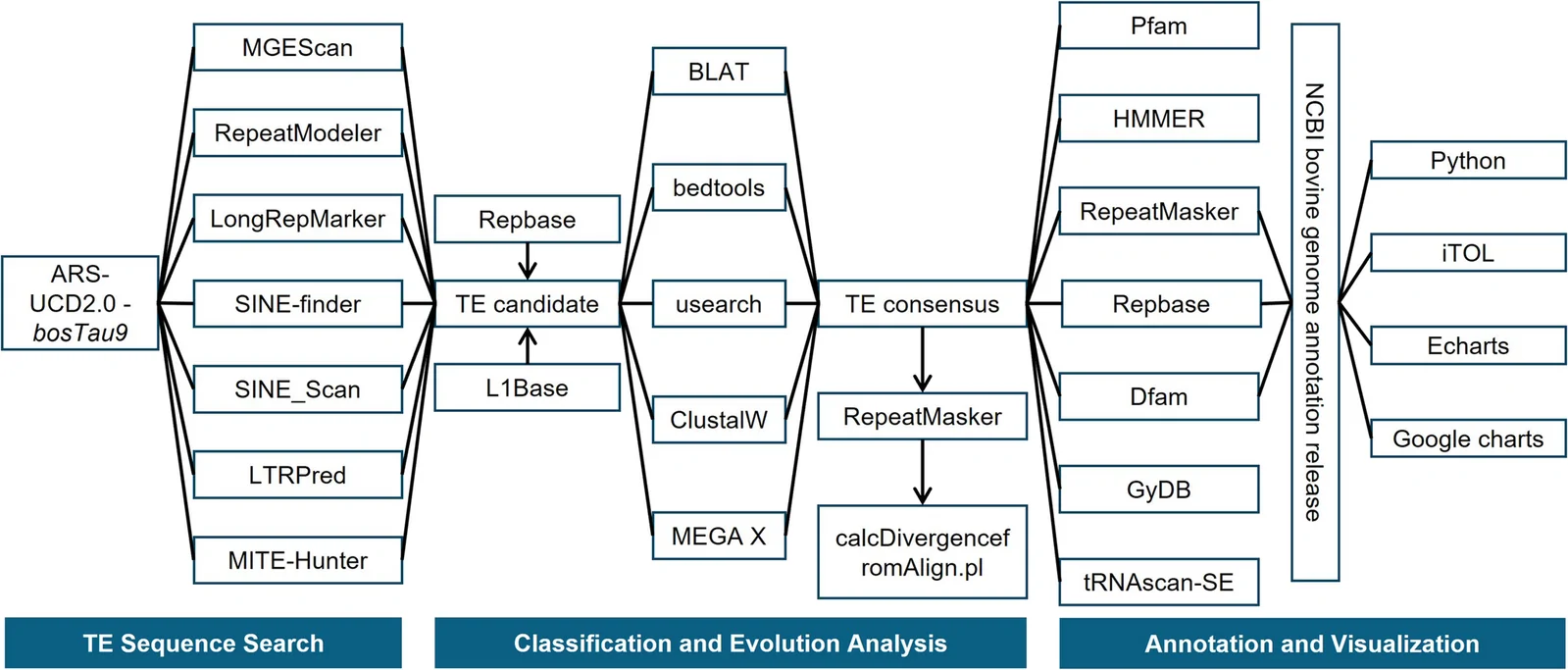
Workflow for retrotransposon identification and classification in the bovine genome

The bovine reference genome (ARS-UCD2.0) was downloaded from the NCBI database (https://www.ncbi.nlm.nih.gov/datasets/genome/GCF_002263795.3/, Accessed in December 2024). We developed a pipeline for the identification of retrotransposons, specifically non-LTR retrotransposons, including LINE and SINE, and the LTR retrotransposons particularly ERV (Fig. 1).

The *de novo* identification of LINEs was performed independently using three tools: MGEScan (version 1.1) (Lee et al. 2016), RepeatModeler (version 2.0.3) (Flynn et al. 2020), and LongRepMarker (version 2.1.2) (Liao et al. 2021). In addition, candidate LINE sequences from the bovine reference genome were downloaded from L1Base and merged with the consensus LINE sequences identified through the de novo analyses. Redundant sequences (defined as those located within 3,000 bp on the same strand) were removed from the merged dataset. LINE consensus sequences shorter than 1 kb were discarded. The remaining sequences were then clustered based on sequence similarity using USEARCH, and clusters containing fewer than 10 LINE sequences were excluded. The clustered LINE sequences were subsequently mapped to the bovine reference genome and extended by 1,000 bp at both the 5′ and 3′ ends. SINEs were identified using four tools, SINE_Scan (version 1.1.1) (Mao and Wang 2017), SINE-finder (version 1.0.1) (Wenke et al. 2011), RepeatModeler (Flynn et al. 2020) and LongRepMarker (Liao et al. 2021). RepeatModeler was run three times to improve sensitivity, and default parameters were used for all tools. Identified SINE sequences were filtered to remove those longer than 1 kb or with fewer than 100 genomic copies. The remaining sequences were mapped to the genome to retrieve the coordinates of each copy, which were then extended by 100 bp at both the 5′ and 3′ ends. Sequence boundaries of both LINEs and SINEs were manually refined through alignment using ClustalW (version 2.1) (Larkin et al. 2007), and only sequences with clearly defined boundaries and target site duplications (TSDs) were retained for downstream analyses. Finally, consensus sequences for each cluster were derived using alignAndCallConsensus.pl script, for LINE and SINE separately.

Identification of LTR elements in the bovine reference genome focused specifically on ERV retrotransposons. Full-length ERVs were first identified using four tools: RepeatModeler (Flynn et al. 2020), LTRPred (version 1.1.0) (Drost 2020), LongRepMarker (Liao et al. 2021), and MGEScan (Lee et al. 2016). The identified ERV sequences were merged, and those shorter than 400 bp were removed. The remaining sequences were translated in all six reading frames using SeqKit (version 2.3.1), and protein domains were annotated using Hmmscan against Hidden Markov Model (HMM) profiles from the Pfam database (Mistry et al. 2021). Sequences were further filtered to retain those with detectable reverse transcriptase regions and longer than 400 bp. The identifiable ERV-related protein domains (e.g. gag, pol and env, etc.) were further confirmed by manual inspection. The filtered ERV sequences were then mapped to the bovine reference genome to determine their genomic coordinates, and each was extended by 5,000 bp at both the 5′ and 3′ ends. Finally, consensus sequences for ERV retrotransposons were generated using the alignAndCallConsensus.pl script.

### Phylogenetic analysis

To account for frequent frameshift mutations and premature stop codons in ancient retrotransposon elements, we performed phylogenetic analysis using the nucleotide sequences of LINEs, SINEs, and the RT regions of ERV elements. Multiple sequence alignments were generated for representative consensus sequences of LINEs, SINEs, and ERV RT regions using ClustalW (version 2.1) (Larkin et al., 2007). Phylogenetic analyses were performed with the IQ-TREE (version 2.0.7). The best-fitting substitution model was selected using ModelFinder Plus. Maximum likelihood trees were inferred, and node support was evaluated using 1,000 ultrafast bootstrap replicates and 1,000 SH-like approximate likelihood ratio test replicates. For comparison and classification purposes, reference RT sequences of known ERV families were retrieved from the NCBI nucleotide database.

### Insertion time estimation

Insertion age estimation was performed by calculating Kimura divergence values (K) using the calcDivergenceFromAlign.pl script, available within the RepeatMasker package (version 4.15). A mean nucleotide substitution rate (r) of 2.00 × 10⁻⁹ mutations per site per year was applied, following estimates reported by Liu et al. (Liu et al. 2006). The insertion time (T) for each element was then calculated using the formula T = K / (2r), as described by Kimura (Kimura 1980). Based on these estimates, retrotransposon families were classified as young (< 2 million years), intermediate (2–40 million years), or old (> 40 million years).

### Annotation of transposon elements in the bovine genome

The gene annotation file for the bovine reference genome (ARS-UCD2.0) was retrieved from the NCBI database (https://www.ncbi.nlm.nih.gov/datasets/genome/GCF_002263795.3/, accessed in December 2024). From this annotation, genomic coordinates were extracted for key non-redundant features, such as protein-coding genes, mRNAs, coding sequences (CDS), 5′ untranslated regions (5’UTRs) and 3’UTRs. Additional gene categories such as long non-coding RNAs (lncRNAs) and pseudogenes were also included. For subsequent analysis, 10 kb regions upstream and downstream of protein-coding and lncRNA genes were appended to their respective coordinates to capture potential regulatory and flanking elements. Genomic sequences corresponding to these regions were extracted using the getfasta function from bedtools (Quinlan 2014). To evaluate TE insertions, overlaps between LINEs, SINEs, and ERV elements and gene features, including their 10 kb flanks, were calculated using bedtools intersect, with a minimum overlap threshold of 10 base pairs. All data processing steps, including sequence retrieval, coordinate refinement, file format conversions, duplicate removal, and identification of TSDs were carried out using custom Python scripts (version 3.7.3). Visualization of results was performed using bar and line charts generated via Google Charts (https://developers.google.com/chart), and pie charts created with ECharts (https://echarts.apache.org/).

### SINE insertion polymorphism detection in Holstein cattle

The polymorphism of BosSINE elements was predicted using our previously published tool TypeSINE with default parameters (Zheng et al. 2025). TypeSINE genotypes SINE insertion polymorphisms from short-read whole-genome sequencing data by jointly detecting reference and non-reference insertions. Published whole-genome sequencing data from 536 Holstein cows were downloaded from 23 BioProjects in the NCBI database (Table S1A). Only BosSINEL elements were used for polymorphism detection as they represent the youngest SINE subfamily with the highest likelihood of ongoing retrotransposition activity. Observations in other species such as pig (Chen et al. 2019) and rabbit (Yang et al. 2021) indicate that older SINE families are largely fixed in the genome. Moreover, TypeSINE was specifically designed for SINE elements, as the larger size of LINEs and ERVs precludes reliable polymorphism detection from short-read sequencing data. Briefly, TypeSINE firstly examined whether BosSINEL loci annotated in the bovine reference genome were present in each individual based on read mapping signals. To reduce potential bias caused by missing insertions in the reference genome, the program also searched for additional BosSINEL insertions using the BosSINEL consensus sequence in each individual genome. The results from reference-based and consensus-based detection were merged to generate the final genotype for each locus in each individual. Each BosSINEL locus was assigned one of three genotypes: 1/1 (all reads spanning the locus support the presence of the insertion), 0/0 (no reads supporting the insertion were detected or non-supporting reads were detected), or 1/0 (both insertion-supporting and non-supporting reads were detected) (Table S1B).

Based on the in silico prediction, 7 loci with predicted insertion polymorphisms were selected for experimental validation by PCR in 16 Canadian Holstein cows (Table S1C). A subset of loci showing polymorphism in the initial screening were further genotyped in a larger cohort of 86 Canadian Holstein cows from 4 different herds. Genomic DNA was extracted from milk somatic cells using the DNeasy Blood & Tissue Kit (Qiagen, Toronto, Ontario, Canada) according to the manufacturer’s instructions. DNA quality and quantity was measured with Quant-iT™ PicoGreen^®^ dsDNA Assay Kit (Life Technologies, Burlington, ON, Canada). PCR amplification in a 25 μLreaction mix containing 1 ng genomic DNA, 10 mM dNTPs, 10 µM each primer, Q5 reaction buffer and Q5 High-Fidelity DNA Polymerase (New England Biolabs, Whitby, Ontario, Canada) was performed on a VeritiPro™ 96 well thermal cycler (Thermo Fisher Scientific, Saint-Laurent, Quebec, Canada). The cycling included an initial denaturation at 98 °C for 30 s, 25 to 35 cycles of denaturation, annealing and extension at 98 °C for 10 s, 63 °C for 30 s and 72 °C for 30 s, and a final extension at 72 °C for 2 min. A touch-down PCR amplification strategy was employed for the successful amplification of two fragments (Table S1C). The PCR products were resolved by 1.5% agarose gel electrophoresis and visualized using nucleic acid gel staining.

### DNA methylation patterns of retrotransposons and association with mastitis

The DNA methylation (DNAm) patterns of retrotransposons were investigated using whole-genome DNA methylation sequencing (WGMS) data previously generated from milk somatic cells from 10 healthy cows and 16 cows with naturally occurring subclinical mastitis caused by *Staphylococcus aureus* (SA) (Wang et al. 2024). The DNAm landscapes around retrotransposons were profiled by aligning all elements by their 5′ ends and visualizing average methylation levels across the body and flanks (± 2 kb) of each element. The methylation level of a retrotransposon was calculated as the mean level of its CpG sites. DNA methylation at each CpG site was quantified using the beta value (β), defined as the ratio of methylated reads to total reads. Only retrotransposons harboring at least 3 CpG sites were retained for subsequent analyses.

To assess potential epigenetic associations with subclinical mastitis, differential methylation analysis was conducted between the SA group and healthy group. The DNAm levels of retrotransposons were compared using the Wilcoxon test, with false discovery rate (FDR) correction for multiple comparisons. Retrotransposons exhibiting significantly altered methylation levels (FDR < 0.05 and absolute methylation difference |∆β| > 10%) in SA samples were highlighted as differentially methylated (DM) retrotransposons. DM retrotransposons were further annotated to the genic regions, including promoters, 5’UTRs, exons, introns and 3’UTRs, which were also intersected with differentially expressed genes identified in the same dataset (Wang et al. 2023a). The differentially expressed genes overlapping with DM retrotransposons were submitted for gene ontology (GO) and KEGG pathways enrichment with ClueGO v2.5.10 (Bindea et al. 2009). The significant enrichment of GO terms and KEGG pathways was defined as having Benjamini-Hochberg adjusted (FDR) p values < 0.05. Significantly enriched GO terms and KEGG pathways were further clustered into functional groups based on kappa score that indicates the similarity of associated genes in the term.

## Results

The *de novo* annotation of major retrotransposons, including LINEs, SINEs, and ERVs, in the bovine reference genome (ARS-UCD2.0) was conducted using a multi-step pipeline, as depicted in Fig. 1. Non-redundant genomic features extracted from the reference genome for the annotation included 29,802 protein-coding genes, 253,275 exons, 20,913 mRNAs, 207,351 CDS, 33,901 5’UTRs, 22,476 3’UTRs, 5,619 lncRNAs and 4,436 pseudogenes. The resulting sequences of LINEs, SINEs, and ERVs were subsequently analyzed for classification, genomic coverage, evolutionary dynamics, structural organization, and genomic and epigenomic functional annotation. Table S2 contains the full set of genomic coordinates for all transposable elements identified in this study.

### *De novo* mining of long interspersed nuclear elements in the bovine genome

A total of 555, 8,265, and 385 LINE sequences were identified using RepeatModeler, LongRepMarker, and MGEScan programs, respectively. These LINE sequences identified through *de novo* prediction were combined with 3,025 bovine LINE sequences obtained from the L1base database. Sequences shorter than 1 kb were discarded, and redundancy was eliminated. The remaining sequences were aligned to the bovine reference genome, and those with a copy number below 10 were excluded. These sequences were extended with flanking regions (1,000 bp upstream and downstream) and submitted for classification. Consensus sequences were constructed for each LINE family, and the TSD sequences, 5’ UTR, 3’ UTR, and coding frames were manually verified for each family.

**Fig. 2:**
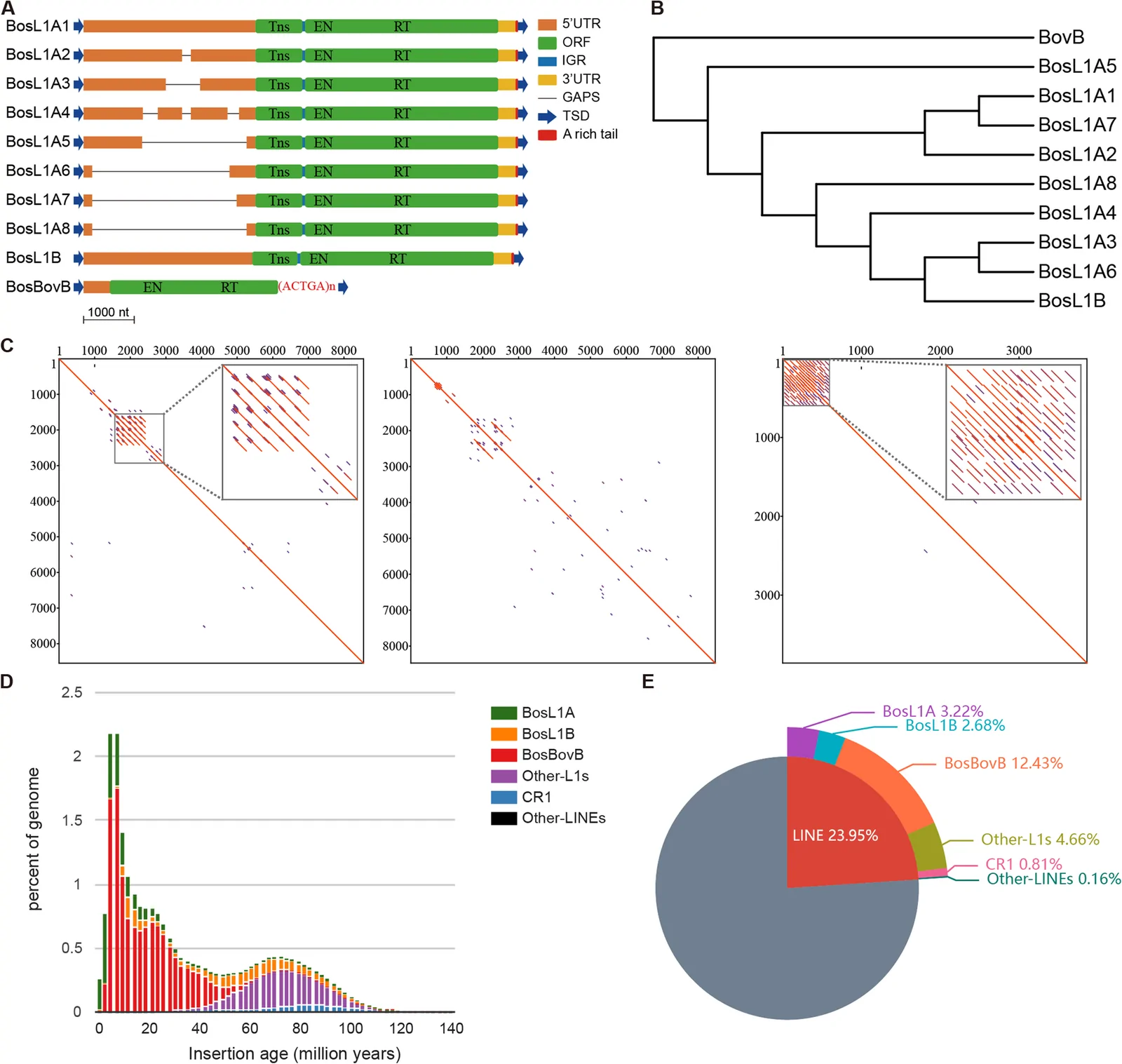
Structure and evolutionary analysis of LINE elements in the bovine genome. (**A**) Structural schematics of major LINE families, including BosL1A subfamilies, BosL1B, and BosBovB. (**B**) Maximum likelihood phylogenetic tree of LINE elements showing three distinct families: BosL1A, BosL1B, and BosBovB. (**C**) Dot plots illustrating structural features of BosL1A1 (left), BosL1B (middle), and BosBovB (right). (**D**) Distribution of estimated insertion ages for LINE elements. (**E**) Proportion of each LINE family represented in the bovine genome. Tns: transposase (transposon-associated enzyme/domain); EN: endonuclease; RT: reverse transcriptase; ORF: open reading frame; GAPS: genomic assembly gaps; TSD: target site duplication; Other-L1: L1 elements that are not BosL1A, BosL1B, and BosBovB; CR1: chicken repeat 1 (CR1) non-LTR retrotransposons; LINE: long interspersed nuclear elements

Ultimately, three LINE families were identified and named: BosL1A, BosL1B, and BosBovB. BosL1A was further divided into eight subfamilies (BosL1A1-BosL1A8) based on structure and IQ-tree analysis (Fig. 2A-B), with consensus sequences for each subfamily provided in Table S3. The total length of the LINE consensus sequences ranged from 3,856 to 8,533 bp. BosL1A and BosL1B displayed typical L1 structures, including a 5’ UTR, 3’ UTR, intergenic regions (IGR), coding frame, and poly(A)-tails, and flanked by TSD sequences. The subfamilies BosL1A1-BosL1A8 contained two complete open reading frames (ORFs) (ORF1 and ORF2). BosL1A1 possessed the most complete structure, with a total length of 8,535 bp, including 3,397 bp 5’ UTR, 927 bp ORF1 (309 aa), 3,819 bp ORF2 (1,273 aa), and 341 bp 3’ UTR (excluding the poly(A)-tail), and was the most recent subfamily, whereas BosL1A2-BosL1A8 exhibited variable deletions in the 5’ UTR (Figure S1 and Table S3). Most open reading frames (ORF1 and ORF2) of BosL1B were incomplete due to accumulated mutations. BosBovB had a distinct structure, measuring only 3,856 bp, with a very short 5’ UTR, lacking ORF1 (Tns) and 3’ UTR-poly(A)-tail, replaced by tandem repeats of (ACTGA)n, as shown in Fig. 2A. Structural comparison revealed that the major differences among BosL1A subfamilies are confined to the 5’ UTR, which also constitutes the key structural feature distinguishing BosL1A from BosL1B (Fig. 2C). In contrast, BosBovB elements are characterized by numerous short repeat sequences at the proximal region of their 5′ end (Fig. 2C).

Evolutionary dynamics indicated that BosL1A and BosBovB exhibit recent and ongoing activities in the genome, dominating evolution over the last 50 million years. BosL1B showed both recent and ancient proliferation, but its activities have largely ceased over the past 10 million years. Other L1s and CR1s represent ancient proliferation, dominating genome evolution between 40 and 120 million years, with their activities halting over 40 million years (Fig. 2D). Neither BosL1A nor BosL1B show significant presence in the genome, occupying only 3.22% and 2.68% of genomic sequences, respectively, while BosBovB occupies 12.43% (Fig. 2E).

### *De novo* mining of short interspersed nuclear elements in the bovine genome

Four software programs were employed for the *de novo* identification of SINE transposons: RepeatModeler, SINE_Scan, LongRepMarker, and SINEfinder. These programs identified 161, 10, 1445, and 9448 SINE sequences, respectively. The identified sequences were merged, and redundant sequences and sequences exceeding 1 kilobase were removed. The remaining sequences were subjected to a BLAST search to determine their copy number and locations in the bovine reference genome. Sequences with fewer than 100 copies in the genome were discarded. The remaining sequences were extended by 100 bp on both sides, and submitted for classification and multiple sequence alignment. Subsequently, the boundaries and TSD sequences were manually inspected for each cluster. Consensus sequences were constructed for each cluster with detectable boundaries and TSD by using the script alignAndCallConsensus.pl, and utilized for further analysis.

**Fig. 3:**
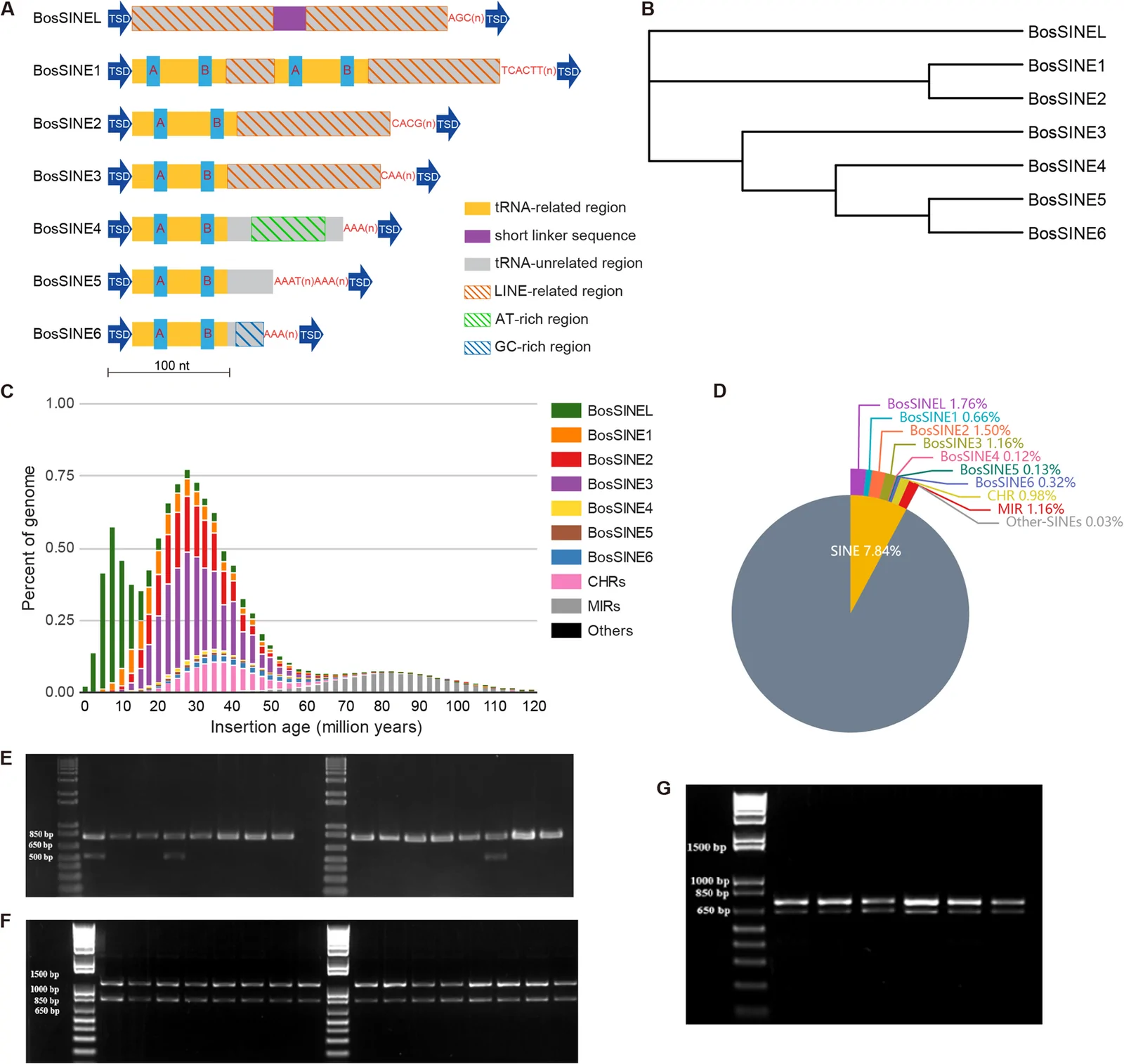
Structure and evolutionary analysis of SINE elements in the bovine genome. (**A**) Structural schematics of SINE families, BosSINE1, and BosSINE1 to BosSINE7. (**B**) Maximum likelihood phylogenetic tree of SINE elements showing distinct families (**C**) Proportion of each SINE family represented in the bovine genome. (**D**) Distribution of estimated insertion ages of SINE elements. (**E**-**G**) Representative agarose gel images showing PCR genotyping results for three loci. (**E**) Locus RTIP2 displays insertion polymorphism with two genotypes detected (1/1 and 1/0), (**F**) locus RTIP3 shows only the heterozygous genotype (1/0) in all tested individuals whereas and (**G**) RTIP6 shows the heterogenous genotype (1/0) present in over 95% of tested individuals. Band patterns correspond to the presence (1) or absence (0) of the BosSINEL insertion at the target locus, where the presence of a band reflects either the insertion (longer fragment) or the non-insertion allele (shorter fragment). CHRs: Chimeric SINE-like element containing mixed repeat sequences; MIRs: mammalian-wide interspersed repeats which forms an ancient SINE family shared across mammals

By aligning and analyzing the consensus sequences, the structural composition of the seven identified sequences were determined and named BosSINEL (276 bp in length), and BosSINE1 to BosSINE6, ranging from 130 to 330 base pairs in length (Fig. 3A-B, Table S3). The BosSINE1 to BosSINE6 elements exhibit the typical structure of SINEs, comprising a head, body, and tail. The head is a tRNA-related region, an evolutionary element derived from a tRNA synthesized by RNA Polymerase III (Vassetzky and Kramerov 2013). BosSINEL does not contain tRNA-related sequences, whereas BosSINE1 contains two such regions. The bodies of BosSINEL and BosSINE1-3 feature a LINE-related region, while the bodies of BosSINE4-6 have a tRNA-unrelated region, characterized by AT or GC-rich sequences (Fig. 3A).

Evolutionary dynamics analysis indicates that BosSINEL is the most recent SINE family, with a substantial number of copies integrated into the genome within the last 2 million years. This suggests that it may still be active in the bovine genome. In contrast, BosSINE1-6 and Chimeric SINE elements (CHR) were predominant in genomic evolution between 5 and 60 million years ago, showing decreased activity in the last 5 million years. SINE/MIRs (mammalian-wide interspersed repeats) dominated between 50 and 120 million years ago, with its activity ceasing in the past 40 million years (Fig. 3C). SINEs constitute approximately 7.84% of the bovine genome (Fig. 3D). The most prevalent family, BosSINEL, comprises 1.76% of the genome, followed by BosSINE2 and BosSINE3, comprising 1.50% and 1.16% of the genome, respectively. Other SINE families each account for less than 1% of the genome. All families, except for BosSINE4, exhibit consensus sequences with a GC content exceeding 50% (Table S3).

Polymorphism analysis using TypeSINE among 536 unique Holstein cows whole genome sequences identified 5,590 polymorphic BosSINEL loci, defined as loci where both non-supporting (0) and supporting (1) alleles were detected with a frequency greater than 10% across individuals (Table S1B). These polymorphic loci accounted for 0.44% of all BosSINEL elements annotated in the bovine reference genome. Among the polymorphic BosSINEL loci detected (*n* = 5590), 4,078 showed all three genotypes (1/1, 1/0, and 0/0), indicating widespread insertion polymorphism in the Holstein population. An additional 1,501 loci displayed two genotypes (1006 with 1/0 and 0/0, 495 with 1/1 and 1/0), whereas only 11 loci showed heterozygous genotype pattern (1/0). These results demonstrate that the youngest BosSINEL elements remain highly polymorphic in the bovine genome, consistent with recent or ongoing retrotransposition activity.

To experimentally validate the in silico predictions, seven polymorphic loci were randomly selected for PCR analysis in a small cohort of 16 cows (Table S1C-D). Three loci showed evidence of insertion polymorphism (Fig. 3E-G, with both heterozygous (1/0) and/or homozygous insertion (1/1) genotypes detected, whereas the remaining four loci showed only the homozygous insertion genotype (1/1) (Figure S2). The three polymorphic loci were subsequently genotyped in a larger cohort of 86 cows (Table S1D). Loci RTIP2 and RTIP6 displayed two genotypes (1/1 and 1/0), with BosSINEL insertion frequencies of 57.56% and 50.58%, respectively (TableS1D). For RTIP3, only the heterozygous genotype (1/0) was detected, resulting in an estimated insertion frequency of 50% (Table S1D).

### *De novo* mining of endogenous retroviruses in the bovine genome

A total of 1,838, 5,286, 36,035, and 1,771 LTR sequences were obtained using the RepeatModeler, LTRPred, LongRepMarker, and MGEScan software tools, respectively. These sequences were merged and redundant sequences were removed. Sequences shorter than 400 bp, those with reverse transcriptase (RT) regions shorter than 400 bp, and sequences containing LINE RT regions were discarded as described in the Methods section. The genomic coordinates of the LTR RT regions were identified using BLAST, and sequences were extended 5,000 bp upstream and downstream to obtain the full-length LTRs. The extracted sequences were used for classification and to construct consensus sequences, as described in the methods. Because ERVs constitute the major and most structurally complete class of LTR retrotransposons in mammalian genomes, further analyses were focused on the annotation and characterization of ERV families.

**Fig. 4:**
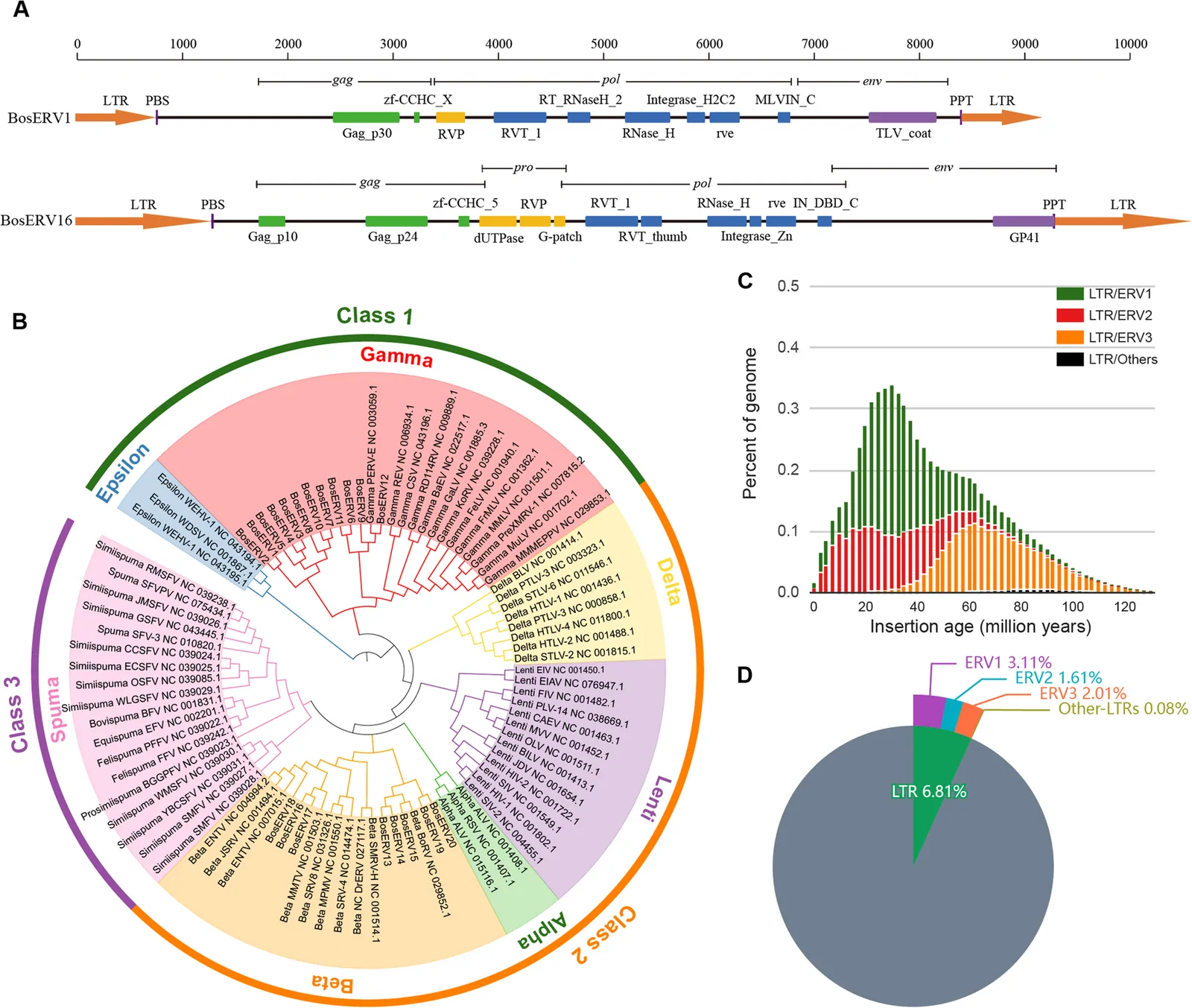
Structure and evolutionary analysis of ERV elements in the bovine genome. (**A**) Structural schematics of BosERV1 and BosERV16 subfamilies. BosERV1 and BosERV16 are shown as representative models of Class I and Class II ERVs, respectively. (**B**) Maximum likelihood phylogenetic tree based on known ERV sequence. (**C**) Estimated insertion ages for LTR elements. (**D**) Proportion of each LTR class represented in the bovine genome. *Gag*: group-specific antigen gene; *Env*: envelope gene; *Pol*: polymerase gene; *Pro*: protease gene; dUTPase: deoxyuridine triphosphatase; EVP: envelope protein (putative); Gag_p10: Gag p10 matrix-related protein; Gag_p24: Gag p24 capsid protein; Gag_p30: Gag p30 capsid-related protein; GP41: envelope glycoprotein 41 subunit; Integrase_H2C2: integrase catalytic core domain (His–His–Cys–Cys motif); Integrase_Xn: integrase N-terminal extension domain; LTR: long terminal repeat; MLVIN_C: Moloney murine leukemia virus integrase C-terminal domain; PBS: primer binding site; PPT: polypurine tract; PPTLTR: polypurine tract–LTR junction region; RNase_H: ribonuclease H domain; RVT_1: reverse transcriptase domain (family 1); RVT_thumb: reverse transcriptase thumb subdomain; rve: integrase core domain (rve family); rveIN_DBD_C: integrase DNA-binding domain (C-terminal, rve family); TLV_coat: human T-lymphotropic virus coat protein domain; zf-CCHC_5: CCHC-type zinc finger domain (variant 5); zf-CCHC_X: CCHC-type zinc finger domain

In total, 20 ERV families were identified based on phylogenetic and structural analyses, and designated BosERV1–BosERV20 (Fig. 4A-B). The bovine ERV families ranged in length from 6,323 to 10,596 bp, with LTRs varying from 341 to 1,291 bp (Table S3). The phylogenetic tree revealed that ERV elements in the bovine genome belong to Class 1 (Gamma genera) and Class 2 (Beta genera). The structure of BosERV1-12, which belong to Class 1, differs from BosERV13-20 in Class 2; BosERV13-20 contains a pro gene coding for dUTPase, which is absent in BosERV1-12. 

Evolutionary dynamic analysis revealed that ERVs have undergone significant differential expansion in the bovine genome (Fig. 4C, Figure S3). From approximately 120 to 40 million years ago, Class III ERVs (ERV3) were predominant in genome evolution, peaking in activity around 60 million years ago and ceasing in the past 40 million years. In contrast, Class I ERVs (ERV1) and Class II ERVs (ERV2) coevolved in the bovine genome over the past 60 million years, followed by a significant recent decrease, indicating very limited current activity (Fig. 4C). Evolutionary dynamic analysis reveals that BosERV1 and BosERV16, belonging to Class I and Class II respectively, are the two youngest families with recent and current activities (< 2 Mya) (Figure S3). BosERV1 has a total length of 9,181 bp, including 762 bp LTRs, and encodes the canonical gag, pol, and env genes required for retroviral replication. In contrast, BosERV16 is shorter (8,225 bp) but contains longer LTRs (1,291 bp) and additionally encodes a pro gene alongside gag, pol, and env. Further analysis of all the elements of BosERV1 and BosERV16 families demonstrated that only four BosERV1 elements (NC_037339.1:37568869–37577998(-), NC_037340.1:74017910–74026489(-), NC_037342.1:78913272–78922417(-), and NC_037357.1:85947479–85956637(+)), and six BosERV16 elements (NC_037345.1:59909148–59919759(-), NC_037351.1:12510497–12521093(+), NC_037353.1:3121324–3131907(-), NC_037357.1:61082980–61093395(+), NW_020190428.1:58135–68721(-), and NW_020190901.1:4726–15123(+)) retain intact structures and complete coding capacity, suggesting that these elements are putatively active. ERV elements constitute a relatively small proportion (6.81%) of the bovine genome, with ERV1, ERV2, and ERV3 occupying 3.11%, 1.61%, and 2.01% of the genome, respectively (Fig. 4D).

### Functional annotation of the bovine mobilome

**Fig. 5:**
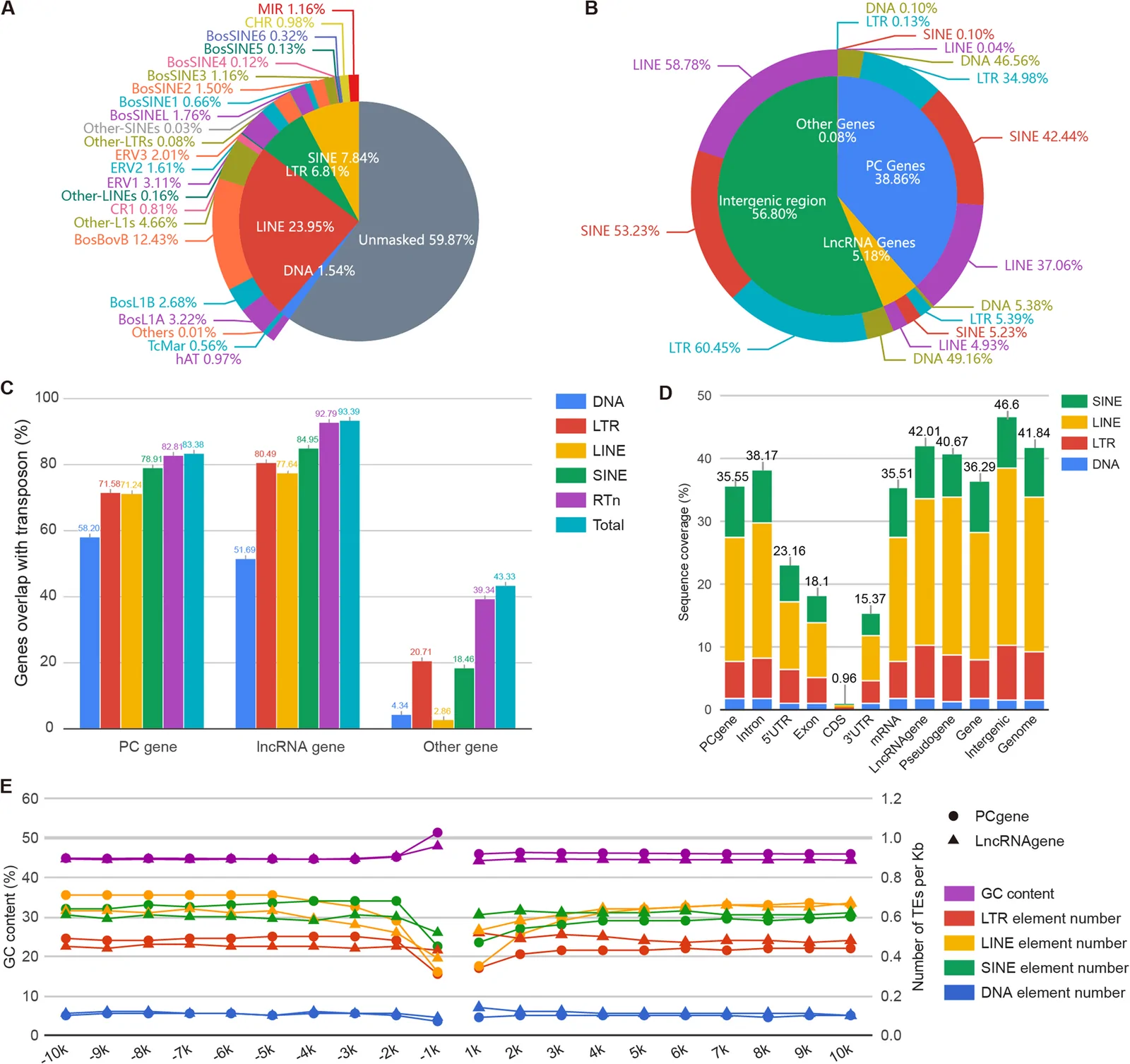
Distribution of transposon elements (TE) in the bovine genome. (**A**) Proportion of TE in the bovine genome. Inner circle indicates the proportion of four classes of transposons annotated in the bovine genome. The outer circle indicates the proportion of families per class. (**B**) The proportion of TEs overlapping with protein-coding genes (PC genes), and long noncoding RNA genes (lncRNA genes). Inside the pie chart represents the proportion of TEs distributed within these regions, whereas outside represents the proportion of different types of TE in each region. (**C**) Proportion of genes overlapped with different types of TE in each PC and lncRNA gene. (**D**) Proportion of genic features overlapped with TEs. (**E**) GC contents (excluding TEs) and TE density in the flank regions of PC genes and lncRNA genes. DNA: DNA transposons; LINE: Long Interspersed Nuclear Elements; SINE: Short Interspersed Nuclear Elements; LTR: Long Terminal Repeat; ERV: Endogenous Retroviruses; RTn: retrotransposons; CDS: coding sequence

Overall, TEs comprise approximately 40% of the bovine genome. LINEs are the most prevalent, constituting 23.95% of the genome, followed by SINEs, which account for about 7.84%. LTRs represent roughly 6.81% of the bovine genome (see Fig. 5A).

The intersection analysis revealed that retrotransposons significantly impacted both lncRNA and protein-coding genes. Specifically, 38.86% of retrotransposons overlapped with protein-coding genes and 5.18% overlapped with lncRNA genes. Among the retrotransposon types, 42.44% of SINEs, 37.06% of LINEs, and 34.98% of LTRs overlapped with protein-coding genes, while approximately 5% of each type overlapped with lncRNA genes, as shown in Fig. 5B. Furthermore, over 80% of protein-coding genes and 90% of lncRNA genes contain TE or retrotransposon insertions. More than 70% of these genes contain sequences derived from each retrotransposon type (LINE, SINE, or LTR), as depicted in Fig. 5C. About 35.55% of protein-coding gene sequences and their introns were derived from TE elements, whereas TE sequences were largely absent in the 5’ and 3’ UTRs and nearly absent in CDS. Moreover, 35.51% of mRNA sequences, 42.01% of lncRNA sequences, and 40.67% of pseudogene sequences originated from TEs, as indicated in Fig. 5D. While CDS, 5’UTRs, and exons of protein-coding genes have higher GC content, they have low TE content (Fig. 5D). TE sequences were largely depleted in regulatory regions (1–3 kb upstream and downstream) of protein-coding genes, lncRNA, and other genes, particularly in upstream (1–2 kb), where high GC content was observed, as illustrated in Fig. 5E and Figure S4.

### DNA methylation pattern of retrotransposons in the bovine genome

**Fig. 6:**
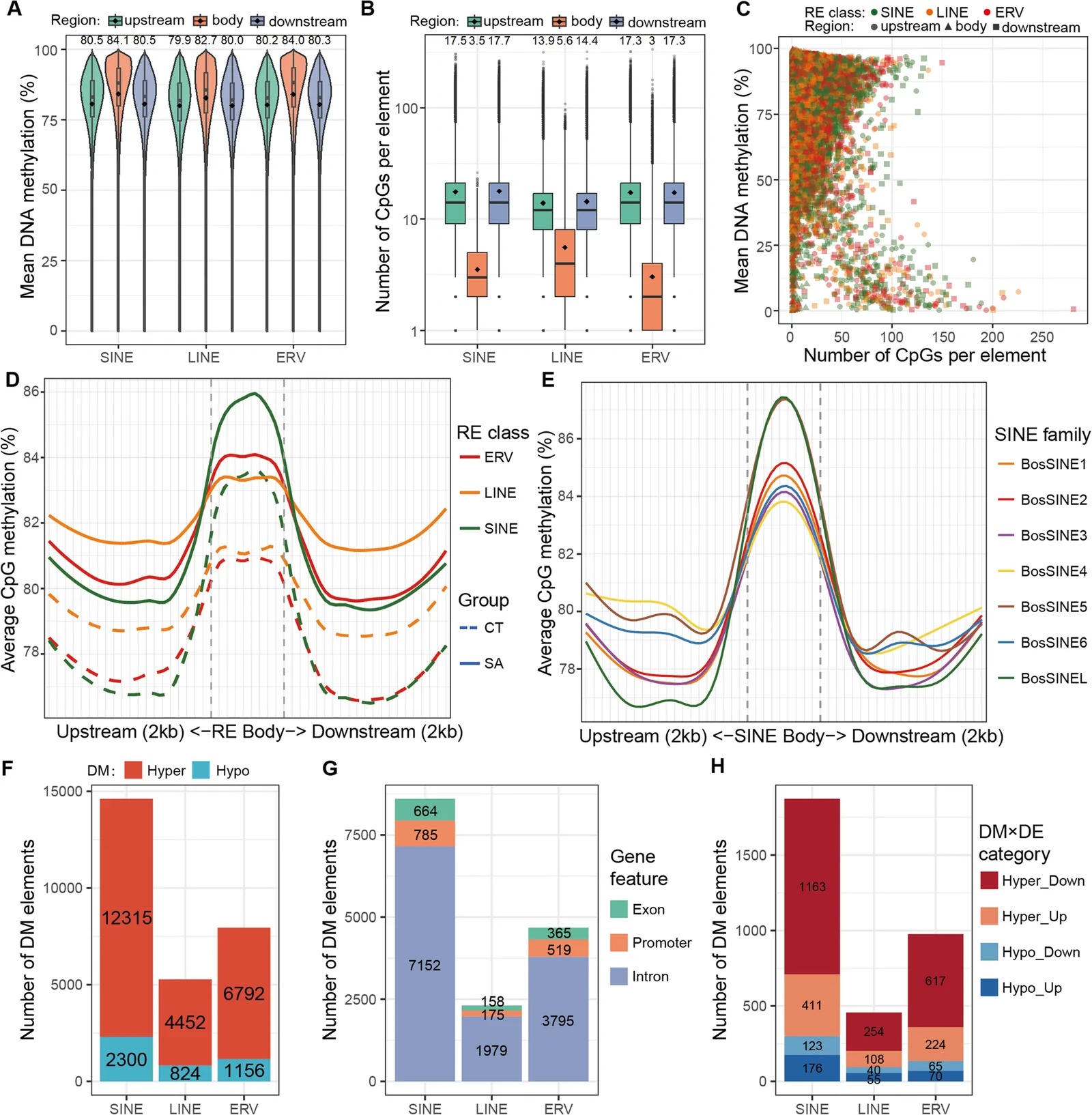
DNA methylation profiles of retrotransposons in bovine somatic cells. (**A**) Average DNA methylation levels of retrotransposons (SINE, LINE, and ERV) and their flanking regions (2 kb upstream and 2 kb downstream) in the bovine milk somatic cells. (**B**) Number of CpG sites within retrotransposons and their flanking regions. (**C**) Distribution of DNA methylation levels and CpG site density across retrotransposons. (**D**) DNA methylation profiles surrounding retrotransposons in healthy control (CT) cows and cows with *Staphylococcus aureus*–induced subclinical mastitis (SA). (**E**) DNA methylation profiles across SINE subfamilies. (**F**) Number of differentially methylated (DM) retrotransposons. “Hyper” and “Hypo” indicate higher or lower DNA methylation in the SA group compared with the CT group. (**G**) Number of DM retrotransposons overlapping genic regions, including promoters, exons, and introns. (H) Number of DM retrotransposons overlapping differentially expressed (DE) genes. “Up” and “Down” indicate genes that are up- or down-regulated in the SA group relative to the CT group. SINE: Short Interspersed Nuclear Elements; LINE: Long Interspersed Nuclear Elements; ERV: Endogenous Retroviruses; RE: retrotransposon.

Retrotransposons showed high DNA methylation levels across the genome in milk somatic cells of dairy cows. On average, they were methylated above 80%, which was slightly higher than the methylation levels observed in their 2 kb upstream and downstream flanking regions. Among the major retrotransposon classes, SINE and ERV elements displayed slightly higher methylation level (84%) than LINE elements (82.7%) (Fig. 6A). Although retrotransposons were highly methylated, they contained fewer CpG sites than their flanking regions. LINE elements had the highest CpG density, with an average of 5.6 CpG sites per element, while SINE and ERV elements averaged 3.5 and 3 CpGs, respectively. However, the flanks of LINE elements had fewer CpG sites than the flanks of SINE and ERV elements (Fig. 6B-C).

Across all retrotransposons, DNA methylation followed a peak-shaped pattern, with the element body showing the highest methylation and methylation decreasing toward both upstream and downstream flanks. This pattern was most pronounced in SINE elements, which had the highest methylation levels within the element body and the lowest in flanking regions compared to LINE and ERV elements (Fig. 6D). Analysis of individual retrotransposon families confirmed this pattern across all SINE families, with the BosSINEL family showing the typical example (Fig. 6E). In contrast, several LINE and ERV families such as BosL1A1, BosL1A2, BosL1A3, BosERV1, BosERV8–10, and BosERV13–18, did not follow this methylation pattern (Figure S5). These families are also among the less abundant groups in the genome (Table S3).

### Retrotransposon DNA methylation changes associated with subclinical mastitis

Cows with subclinical *Staphylococcus aureus* mastitis (SA) had globally higher retrotransposon methylation level than healthy controls (HC) (Fig. 6D). A comparison of methylation profiles revealed widespread differential methylation, with significant changes (FDR < 0.05 and absolute methylation difference |Δβ| > 10%) in 14,615 SINEs, 5,276 LINEs, and 7,948 ERV elements (Table S4). Most differentially methylated (DM) retrotransposons were hypermethylated (i.e., higher methylation in the SA group compared with healthy controls) in the SA group. Specifically, 12,315 SINEs, 4,452 LINEs, and 6,792 ERVs were hypermethylated, representing more than 84% of DM elements in each class (Fig. 6F). Approximately half of the DM retrotransposons overlapped with genes, including 8,232 SINEs, 2,236 LINEs, and 4,451 ERVs, and most overlaps occurred within introns (Fig. 6G). These intronic changes highlight potential effects on gene regulation.

The distribution of DM elements varied notably across retrotransposon families. Among them SINEs, BosSINE3, BosSINE2, and BosSINEL contained the largest numbers of DM elements (4,292; 4,273; and 3,719, respectively), representing 6.98%, 4.40%, and 2.24% of all family members with at least three CpG sites (Table S4A). These proportions were much higher than in other SINE families; for example, BosSINE1 had only 1,038 DM elements, and most other families had fewer than 1,000. For LINEs, BosBovB contributed 3,062 DM elements (1.04% of all BosBovB copies). Although BosL1 families had fewer DM elements overall, the proportion of DM elements within each family was higher. BosL1B, for instance, had 1,232 DM elements (2.88%), and several BosL1A subfamilies showed comparable proportions, including BosL1A3 (356 of 9,271; 3.84%), BosL1A6 (354 of 16,504; 2.14%), and BosL1A8 (88 of 5,734; 1.53%). Among ERVs, BosERV12 had the largest number of DM elements (3,759; 3.30%), while BosERV10 had only 34 DM elements but showed the highest percentage (19.0%) relative to its total copy number.

### Epigenetic links between retrotransposon methylation and gene expression in cows with subclinical mastitis

To assess how DNA methylation changes in retrotransposons may relate to gene expression, we mapped DM retrotransposons to differentially expressed (DE) genes. The DE genes were reported previously (Wang et al. 2024). In total, 1,873 SINEs, 457 LINEs, and 976 ERVs overlapped with DE genes (Table S5). Most of the DM retrotransposons (about 60%) were hypermethylated and overlapped with down-regulated genes, while about 20% were hypomethylated and overlapped with up-regulated genes (Fig. 6H). Fisher’s exact tests confirmed that this inverse association was highly significant for all three retrotransposon classes (SINE: OR = 4.05, *p* < 0.0001; LINE: OR = 3.23, *p* < 0.0001; ERV: OR = 2.97, *p* < 0.0001), indicating that the observed pattern is unlikely to reflect random genomic baseline features. These patterns suggest a directional relationship between DNA methylation and gene expression. More than 85% of the overlapping DM retrotransposons were located within introns of DE genes, including 1,652 of 1,873 SINEs (88.2%), 398 of 457 LINEs (87.1%), and 865 of 976 ERVs (88.6%). Only a small fraction overlapped with promoters or exons, but most of them showed inverse relationships with gene expression (Table S5A–C, Figure S6). For example, among the 92 DM SINEs located in the promoters of DE genes, 57 hypermethylated elements overlapped with down-regulated genes, while 15 hypomethylated elements overlapped with up-regulated genes. These promoter-associated hypermethylated SINEs were mainly from BosSINEL (*n* = 18), BosSINE2 (*n* = 17), and BosSINE3 (*n* = 15). Similar trends were observed for ERVs and LINEs. For ERVs, 32 hypermethylated elements were found in promoters of down-regulated genes, compared with only seven hypomethylated ERVs in promoters of up-regulated genes. For LINEs, 11 of 26 promoter-overlapping DM elements were hypermethylated and associated with down-regulated genes.

Some DE genes contained multiple DM retrotransposons, indicating potential combinatorial regulatory effects (Table S5D). For instance, *PCDH9* carried 20 DM retrotransposons (9 SINEs, 6 LINEs, 5 ERVs), all within introns. Other genes, such as *DLG2*, *SHANK2*, *AGAP1*, and *CTNNA3*, each harbored more than 15 intronic DM retrotransposons. Several genes also carried DM retrotransposons in their exons. For example, the down-regulated gene *SH3BP4* contained nine hypermethylated SINEs and eight hypermethylated ERVs in exonic regions. Another down-regulated gene, *KZAN* overlapped with 16 hypermethylated retrotransposons (7 SINEs, 2 LINEs, and 7 ERVs), including several located in exons. These examples highlight that methylation changes within retrotransposons may cluster within specific genes and potentially influence their transcription. Together, these results suggest that DM retrotransposons possibly are not randomly distributed. Instead, those enriched within DE genes may display methylation–expression relationships consistent with regulatory involvement. This pattern supports a potential epigenetic network linking retrotransposon methylation, gene expression, and host responses to *S. aureus* subclinical mastitis.

To explore this network further, we performed functional enrichment analysis of the 1,365 DE genes that overlapped with DM retrotransposons. These genes were enriched in 36 KEGG pathways and 989 GO terms, including 823 Biological Process, 80 Cellular Component, and 86 Molecular Function terms. Clustering based on shared gene sets produced 270 functional groups. Many of these groups were strongly related to immune function. The largest cluster included 139 Biological Process terms and one KEGG pathway, such as cell development (GO:0048468), leukocyte differentiation and activation (GO:0002521 and GO:0045321), regulation of immune system process (GO:0002682), and Inflammatory bowel disease, among others (KEGG:05321) (see “Group270” in Table S5E). These annotations are primarily associated with immune cell development, proliferation, differentiation, and activation, as well as broader regulation of immune processes. These findings highlight the strong involvement of immune pathways in the host response to subclinical mastitis. The second-largest cluster (49 Biological Process terms and 2 Cellular Component terms) was enriched for transmembrane transport functions. These included transmembrane transporter activity, metal ion transport, calcium ion import, and general ion homeostasis. Such processes help maintain ionic balance, regulate intracellular signaling, and support inflammatory responses, all of which are essential during infection. Additional enriched terms involved organ morphogenesis and tissue development, including muscle, epithelium, blood vessels, heart, and neuron projection development. These may relate to tissue remodeling and systemic physiological changes observed in cows experiencing subclinical mastitis. Overall, the enrichment of immune and ion transport pathways among DE genes containing DM retrotransposons supports the idea that epigenetic regulation of retrotransposons may help shape cellular responses to *S. aureus* infection. These coordinated methylation and expression changes point to a broader regulatory role for retrotransposons in mastitis susceptibility and host defense.

## Discussion

Previous studies, which sometimes over- or underestimated genomic repeat content due to limitations in assembly quality, reported that the bovine mobilome constitutes approximately 46.5% of the genome (Adelson et al. 2009). In this study, we performed a systematic *de novo* identification, classification, and reannotation of bovine mobilome components, including LINEs, SINEs, and LTR retrotransposons. We found that the mobilome, dominated by retrotransposons, comprises approximately 40% of the bovine genome. This proportion is highly similar to that reported in other ungulates such as Snow sheep (*Ovis nivicola*, ~ 41.5%) (Upadhyay et al. 2020), pig (*Sus scrofa*, ~ 40.7%) (Chen et al. 2019), and rabbit (*Oryctolagus cuniculus*, ~ 41.6%) (Yang et al. 2021). It is substantially higher than in Bactrian and dromedary camels (~ 32%) (Ibrahim et al. 2021), but lower than in Pashmina goat, mountain goat, water buffalo, wild yak, and horse (range: ~45–49%) (Low et al. 2019; Liu et al. 2020, 2025; Martchenko et al. 2020; Li et al. 2025). While total TE content varies among these species, the relative contributions of different classes also show distinct patterns. In the bovine genome, LINEs are the largest fraction at ~ 24%, a proportion similar to that in horse, Bactrian camel, dromedary camel, and pig (~ 18–20%), but substantially lower than in snow sheep, Pashmina goat, mountain goat, water buffalo, and wild yak (~ 25–40%). Bovine SINEs constitute ~ 8% of the genome, similar to the levels in horse and water buffalo (7–8%), lower than in goat, yak, pig, and rabbit (10–19%), but higher than in snow sheep and camels (2–4%). LTR retrotransposons account for ~ 7% of the cattle genome, a level comparable to that in mountain goat, wild yak, horse, camels, and pig (5–8%), higher than in snow sheep, Pashmina goat, and rabbit (3–4%), but lower than in water buffalo (13.54). Collectively, these findings highlight both conserved features and species-specific variations in TE composition across livestock genomes, offering valuable insights into their evolutionary histories and potential functional significance for genome biology and selective breeding.

In other mammalian species, active “modern” endogenous retroviruses (ERVs), which have integrated post-speciation and are closely related to exogenous viruses, have been identified (e.g., KoRV in koalas, JSRV in sheep, ERV6s in pig and OcuERV1 in rabbit) (Sharp and DeMartini 2003; Chen et al. 2019; Yang et al. 2021; Yu et al. 2025). By using multiple software tools, we identified, structurally characterized and classified RE classes, including 3 LINE families (BosL1A [further subdivided into BosL1A1 to BosL1A8], BosL1B and BosBovB), 7 SINE families (BosSINEL, BosSINE1 to BosSINE6), 20 ERV families (BosERV1–BosERV20) and others. Some of these elements were newly detected through our careful analyses and all elements were named based on their structural characterization for easy reference. Furthermore, our evolutionary and structural analyses revealed distinct activity patterns and identified three autonomous retrotransposons, BosL1A1 (8,535 bp), BosERV1 (9,181 bp), and BosERV16 (8,225 bp), and one non-autonomous element, BosSINEL (276 bp), as the most recently active and potentially still transposition-competent families. BosERV1 and BosERV16 (as a type of LTR retrotransposons) exhibit complete structures and encode functional proteins necessary for integration, hallmarks of “modern” ERVs. BosL1A1 (a number of BosL1A family of non-LTR retrotransposon), which shows evidence of recent insertion, has a complete structure with intact ORF1 and ORF2 coding sequences, indicating it is putatively active and capable of mobilizing itself as well as the non-autonomous BosSINEL elements. Sequence divergence (K) estimates suggest that BosL1A1, BosERV1, BosERV16, and BosSINEL have remained active within the last 2 million years. These results establish retrotransposons as genomic parasites that have significantly shaped bovine genome evolution and are likely drivers of ongoing genomic diversification.

We adapted TypeSINE to predict BosSINEL polymorphisms in 536 Holstein cattle and identified that ~0.44% of all BosSINEL elements annotated in the bovine reference genome are polymorphic in Holstein cattle. The observed polymorphism of these BosSINEL loci is consistent with recent or ongoing retrotransposition activity; however, broader analyses are required to determine their stability across populations and potential breed-specific distribution patterns. In particular, large-scale studies across diverse cattle populations will be important to characterize the distribution of these insertions, assess their evolutionary stability across lineages, and evaluate whether any loci are associated with functional or regulatory effects. Such efforts will be essential to fully resolve the evolutionary dynamics of young SINE subfamilies in the bovine genome and to assess their potential biological relevance.

Retrotransposons are not randomly distributed across the bovine genome. Although they occupy a substantial fraction of genic regions, they are markedly depleted from protein-coding exons, untranslated regions, and promoter-proximal regulatory sequences. This pattern is consistent with strong purifying selection against insertions that disrupt transcription, mRNA processing, or protein structure, a trend widely observed across mammalian genomes (Goerner-Potvin and Bourque 2018; Ilık et al. 2025). The sharp reduction in TE occupancy in the first 1 kb upstream of genes, which are usually enriched for CpG islands and have high GC content, likely reflects a combination of insertion bias and functional constraint. GC-rich promoters are essential transcriptional hubs, and retrotransposons inserting into these sites can interfere with transcription factor binding or chromatin accessibility, resulting in rapid evolutionary removal (Sundaram et al. 2014). At the same time, the pervasive presence of TE sequence within introns and lncRNA loci highlights their long-term contribution to genomic regulatory innovation. A previous study showed that mammalian TEs supply regulatory motifs, splice junctions, promoters, and enhancers that become co-opted into host regulatory networks (Chuong et al. 2017). Similar exaptation events have been reported in livestock, including TE-derived enhancers affecting immune and metabolic pathways in pigs and sheep (Chen et al. 2019; Upadhyay et al. 2020). Such mechanisms are likely operating in cattle as well, particularly within gene-rich intronic regions permissive to gene regulatory innovation.

The importance of DNA methylation in controlling retrotransposons is well established across mammals, and our data provide a genome-wide view of this regulatory layer in the bovine genome. Retrotransposon activity is closely governed by epigenetic control, and their genomic distribution makes them primary targets of repressive DNA methylation and heterochromatin deposition (Jansz 2019). This silencing is clearly reflected in the bovine genome: across all major classes, retrotransposons are heavily methylated in milk somatic cells (average > 80%), mirroring patterns observed across human and mouse tissues where methylation is a major barrier to TE activation (Law and Jacobsen 2010; Deniz et al. 2019). The characteristic “peak-shaped” methylation profile (maximal methylation across element bodies tapering into flanks) observed likely reflects KRAB–ZFP/KAP1-mediated nucleation and spreading of heterochromatin (Rowe et al. 2013; Ecco et al. 2016), with SINEs showing the tightest body-restricted pattern due to their smaller size and reliance on host machinery. In contrast, several low-copy LINE and ERV families deviate from this canonical profile, similar to dynamically regulated families described in pigs and humans (Bourque et al. 2018; Chen et al. 2019). Together, these observations reinforce that retrotransposon impact depends not only on genomic placement but also on their epigenetic state: loss or relaxation of methylation can unmask latent regulatory activity and modulate nearby genes, especially those involved in immunity and development (Chuong et al. 2016).

The widespread and predominantly intronic differential methylation of retrotransposons associated with subclinical *Staphylococcus aureus* mastitis in this study suggests that retrotransposon silencing is not static but dynamically modulated by immune challenge. The strong skew toward hypermethylation in infected cows, impacting more than 80% of significantly altered SINEs, LINEs, and ERVs, suggests active reinforcement of host repression pathways. Comparable infection-associated tightening of TE silencing has been reported in other mammals, where inflammatory and interferon signaling reshapes chromatin and increases DNA methylation at transposable elements (Gázquez-Gutiérrez et al. 2021; Sorek et al. 2022; Wang et al. 2023b, 2024). These findings support the view that TE methylation forms part of a genome-defense program engaged during immune challenge. Notably, nearly half of DM retrotransposons overlapped with genes, and the enrichment of DM retrotransposon insertions within introns of immune-responsive genes raises the possibility that retrotransposons mediate context-dependent regulatory fine-tuning. Intronic retrotransposons are increasingly recognized as reservoirs of latent enhancers and transcription factor binding sites that can modify splicing, transcriptional elongation, and promoter–enhancer communication when epigenetic repression is relaxed (Sundaram et al. 2014; Chuong et al. 2017).

The clear inverse relationship between methylation state and gene expression—hypermethylated retrotransposons predominantly linked to down-regulated genes, and hypomethylated elements associated with up-regulated genes—aligns with known functional roles of DNA methylation. In promoters and first introns, DNA methylation can directly attenuate transcription factor recruitment and block enhancer–promoter contact (Schmitz et al. 2013; Anastasiadi et al. 2018). The enrichment of promoter-overlapping DM retrotransposons from relatively young families, such as BosSINEL and BosL1A, mirrors findings in humans showing that recent TE insertions are disproportionately associated with immune-inducible regulatory activity (Chuong et al. 2016). For instance, specific young TE subfamilies have been shown to harbour binding motifs for inflammatory transcription factors (e.g., NF-κB and AP-1) and to function as stimulus-responsive enhancers, supporting the potential for lineage-specific elements to modulate immune regulation (Bogdan et al. 2020). Furthermore, genes harboring clusters of DM retrotransposons, including immune-related loci such as *PCDH9*, *DLG2* or *CTNNA3*, suggest additive or combinatorial epigenetic effects, a phenomenon observed in cancer epigenomes and immune cell activation states (Deniz et al. 2019). Functional enrichment of DE genes containing DM retrotransposons reinforces the biological relevance of these epigenetic perturbations. Immune signaling, cellular activation, and ion transport, which are all hallmarks of mammary gland inflammatory responses, were prominently overrepresented. In other livestock, TE-derived regulatory elements have been shown to modulate host defense and metabolic pathways (Chen et al. 2019; Upadhyay et al. 2020), and our findings suggest similar rewiring may occur in cattle. Taken together, these patterns support an emerging view that retrotransposons function as both genomic liabilities and regulatory reservoirs: normally constrained by DNA methylation, but capable of contributing to transcriptional plasticity when epigenetic barriers shift during infection.

Despite providing new insight into bovine retrotransposon diversity and epigenetic regulation, several limitations should be acknowledged, together with opportunities for future work. First, although our evolutionary analysis highlights multiple young retrotransposon families that may retain mobilization potential, this inference remains computational. We assessed SINE insertion polymorphisms and validated a subset of loci by PCR, supporting their polymorphic nature. However, validation remains limited in scale, and broader functional and population-level analyses will be required to confirm ongoing activity and clarify their biological and evolutionary significance. Second, our DNA methylation profiling was conducted in milk somatic cells, which consist of a heterogeneous mixture of epithelial and immune cell types rather than a single cell population. This may represent a positive aspect, as it reflects the physiological cellular composition present during mastitis. However, because retrotransposon regulation and DNA methylation are highly tissue- and cell-type-specific, the use of mixed-cell samples limits our ability to determine the cellular origin of the observed methylation changes. Consequently, we cannot exclude the possibility that compositional shifts in cell types between healthy and mastitic samples may contribute to the observed differences in retrotransposon methylation. Future studies using purified cell populations or additional tissues will be required to determine whether the patterns observed here reflect cell-type-specific regulation or more systemic responses. Thirdly, although our methylation analysis was based on uniquely mapped reads from a standard WGBS pipeline, we recognize that mappability bias in repetitive regions, a well-known challenge in short-read epigenomic studies of transposable elements, may have affected per-element methylation estimates. However, our analysis procedure requiring a minimum of three CpG sites per retrotransposon element and a mean β value across all CpGs within each element, reduced the influence of individual spurious measurements in this study. Finally, although associations between retrotransposons, DNA methylation, gene expression, and mastitis susceptibility are compelling, the mechanistic directionality remains unresolved. It is unclear whether retrotransposon hypermethylation acts primarily as a host defense strategy to silence inducible regulatory elements, or whether altered retrotransposon methylation itself perturbs gene networks involved in immunity and infection. Integrating long-read sequencing, nascent transcription assays, CRISPR-based perturbations, and single-cell epigenomics will be critical technical steps toward disentangling these causal relationships. Collectively, such efforts will advance understanding of how retrotransposons contribute to bovine health and may ultimately support the development of epigenetic or genomic markers for mastitis resistance.

## Conclusion

In summary, this study provides the first integrated view of retrotransposon diversity, evolutionary history, and epigenetic regulation in the bovine genome, and reveals how these elements respond to subclinical *Staphylococcus aureus* mastitis. We show that multiple young retrotransposon families hold the potential to continue to shape genome structure. We found that subclinical *Staphylococcus aureus* mastitis is accompanied by widespread hypermethylation across retrotransposon families in milk somatic cells, reflecting strengthened epigenetic repression. These epigenetic shifts intersect extensively with host gene networks, particularly those involved in immunity and cellular homeostasis, suggesting that retrotransposons participate in coordinated regulatory responses to infection. Together, our findings highlight retrotransposons as dynamic components of the bovine genome with potential relevance to disease susceptibility and provide a foundation for future mechanistic and functional work.

## Supplementary information

Below is the link to the electronic supplementary material.

Supplementary Material 1 (XLSX 9.34 MB)

Supplementary Material 2 (BED 68 MB)

Supplementary Material 3 (XLSX 86.1 KB)

Supplementary Material 4 (XLSX 2.76 MB)

Supplementary Material 5 (XLSX 869 KB)

Supplementary Material 6 (PDF 843 KB)

## Data Availability

All data supporting the findings of this study are available within the paper and its Supplementary Information.
