# Supplementary material for: A comprehensive map of the bovine mobilome and their epigenetic regulation of mastitis
Source: Funct Integr Genomics. 2026 Sep 16;26(1):266. doi: 10.1007/s10142-026-01970-5 (PMC13582161; doi:10.1007/s10142-026-01970-5)
Supplement: Supplementary file 1 [file 10142_2026_1970_MOESM6_ESM.pdf]

Supplementary Figures

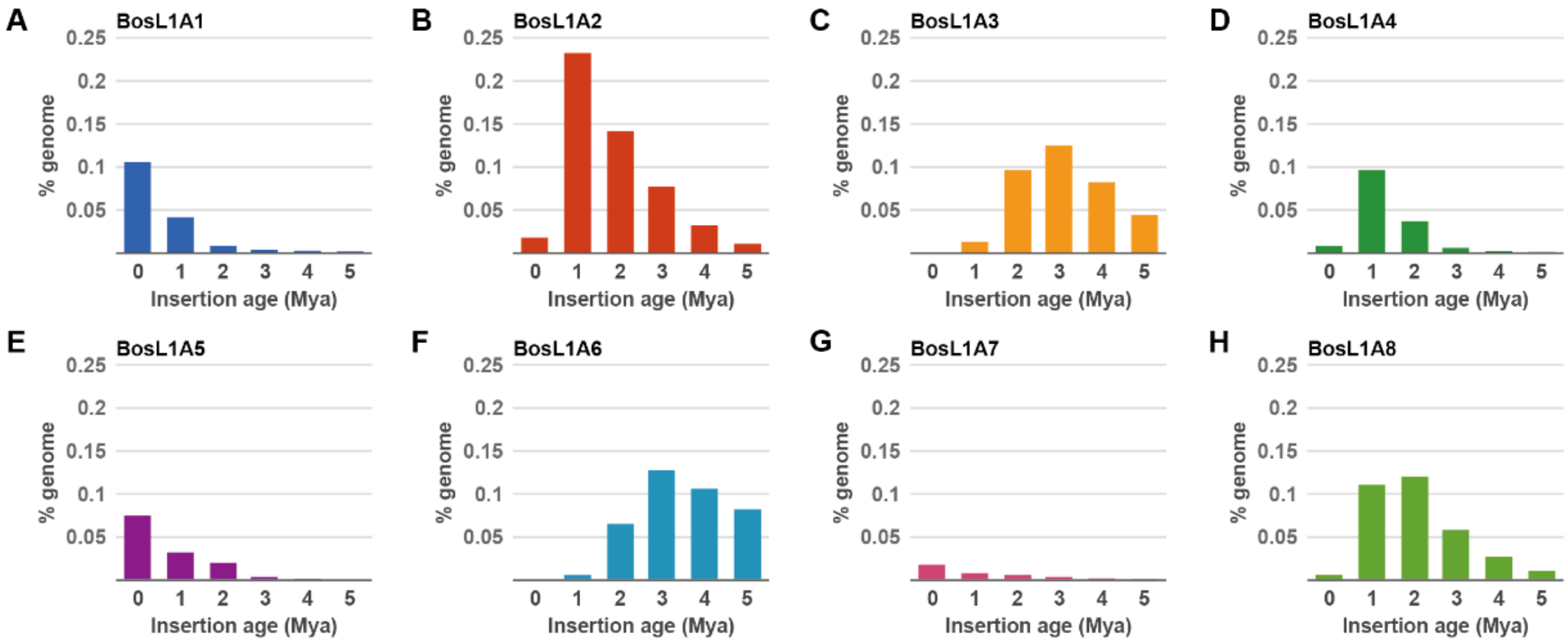

Figure S1 Estimated insertion ages for BosL1A subfamilies. The x-axis represents insertion age in million years (Mya), and the y-axis indicates the percentage of the bovine genome occupied by each BosL1A subfamily.

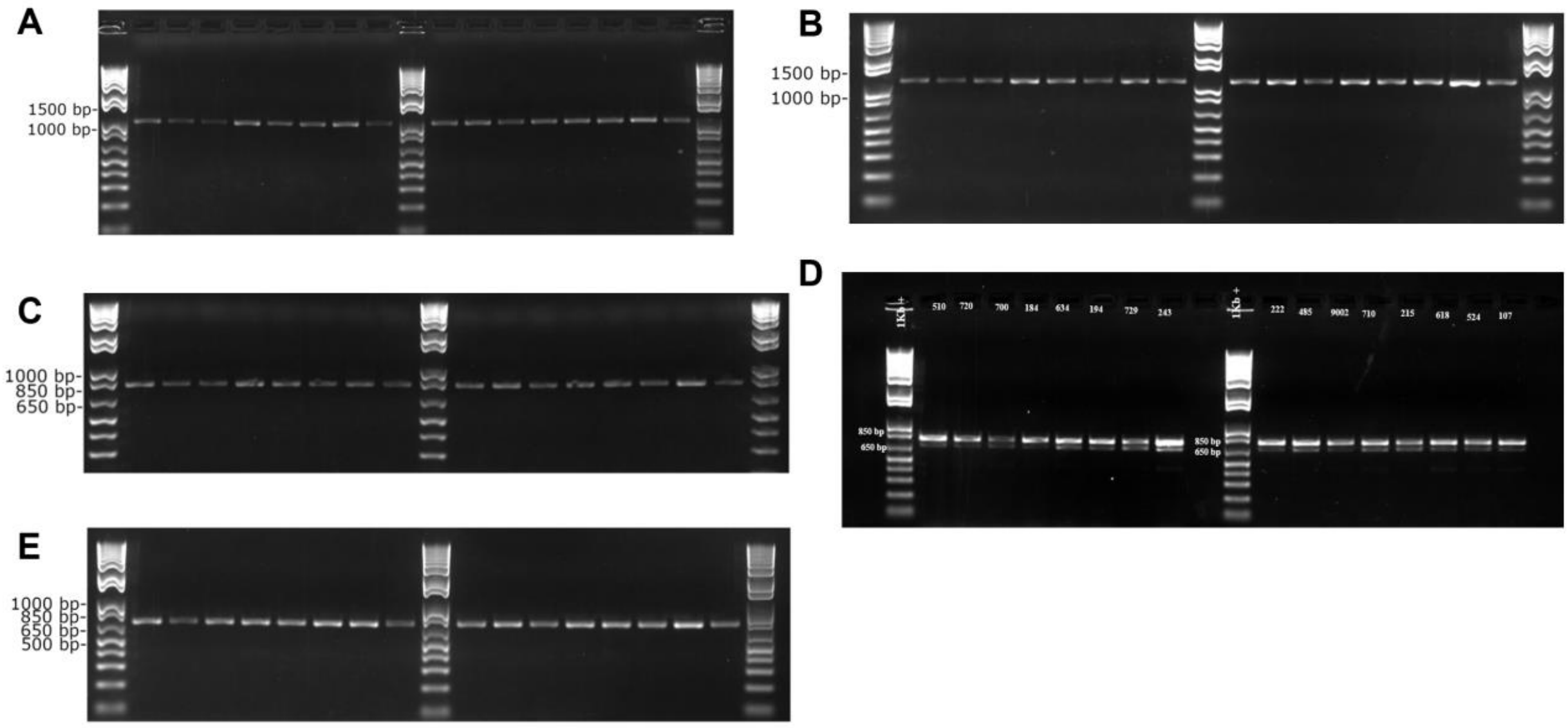

Figure S2 PCR validation of BosSINEL insertion polymorphisms predicted by TypeSINE across 5 genomic loci in Holstein cows. (A): RTIP1; (B): RTIP4; (C): RTIP5; (D): RTIP6; (E): RTIP7. Representative agarose gel images are shown for each locus. Only a subset of the tested samples is displayed here, while the complete genotyping results for all individuals are provided in Table S1D.

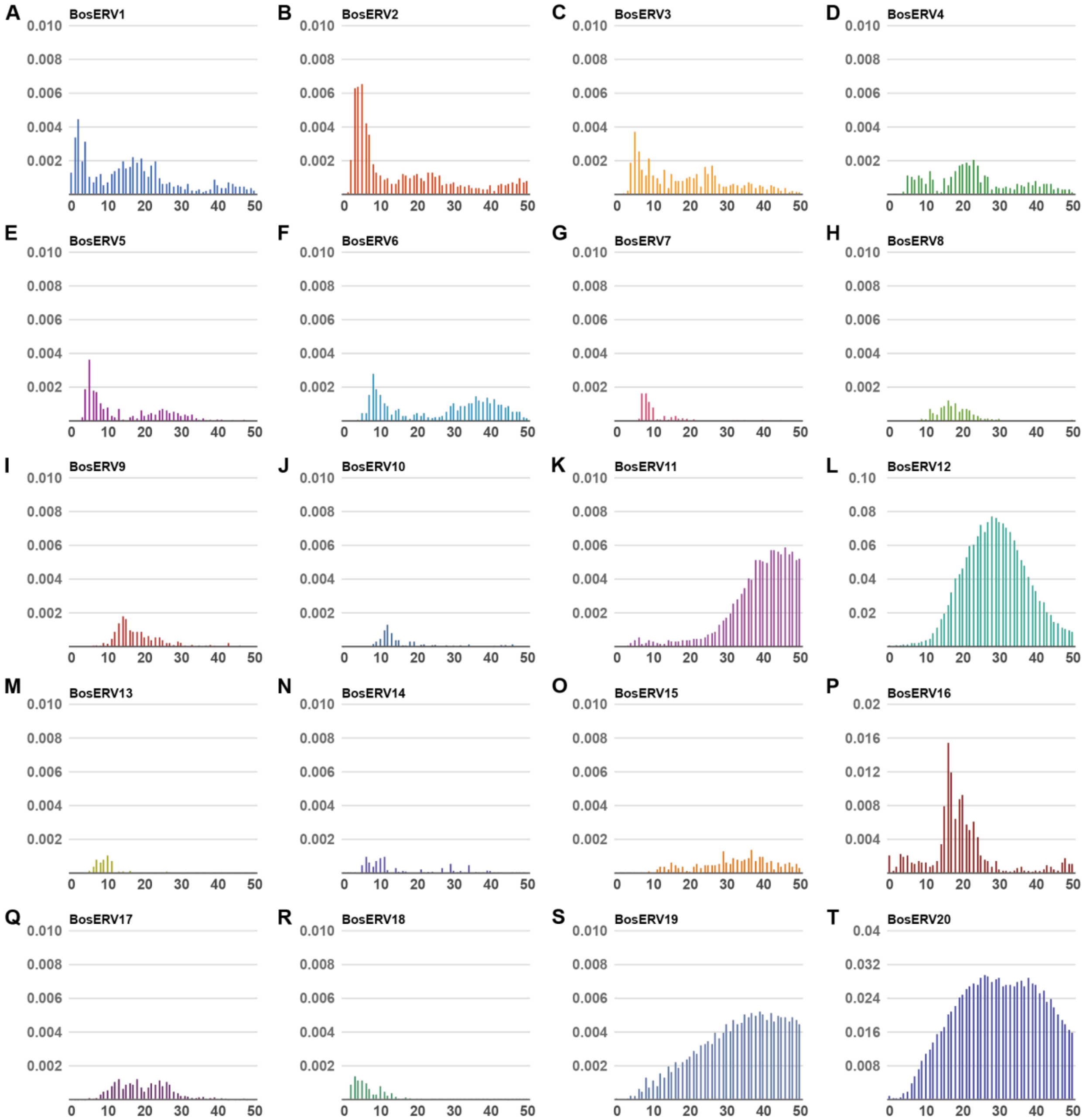

Figure S3 Estimated insertion ages for BosERV retrotransposon subfamilies. The x-axis represents insertion age in million years (Mya), and the y-axis indicates the percentage of the bovine genome occupied by each BosERV subfamily.

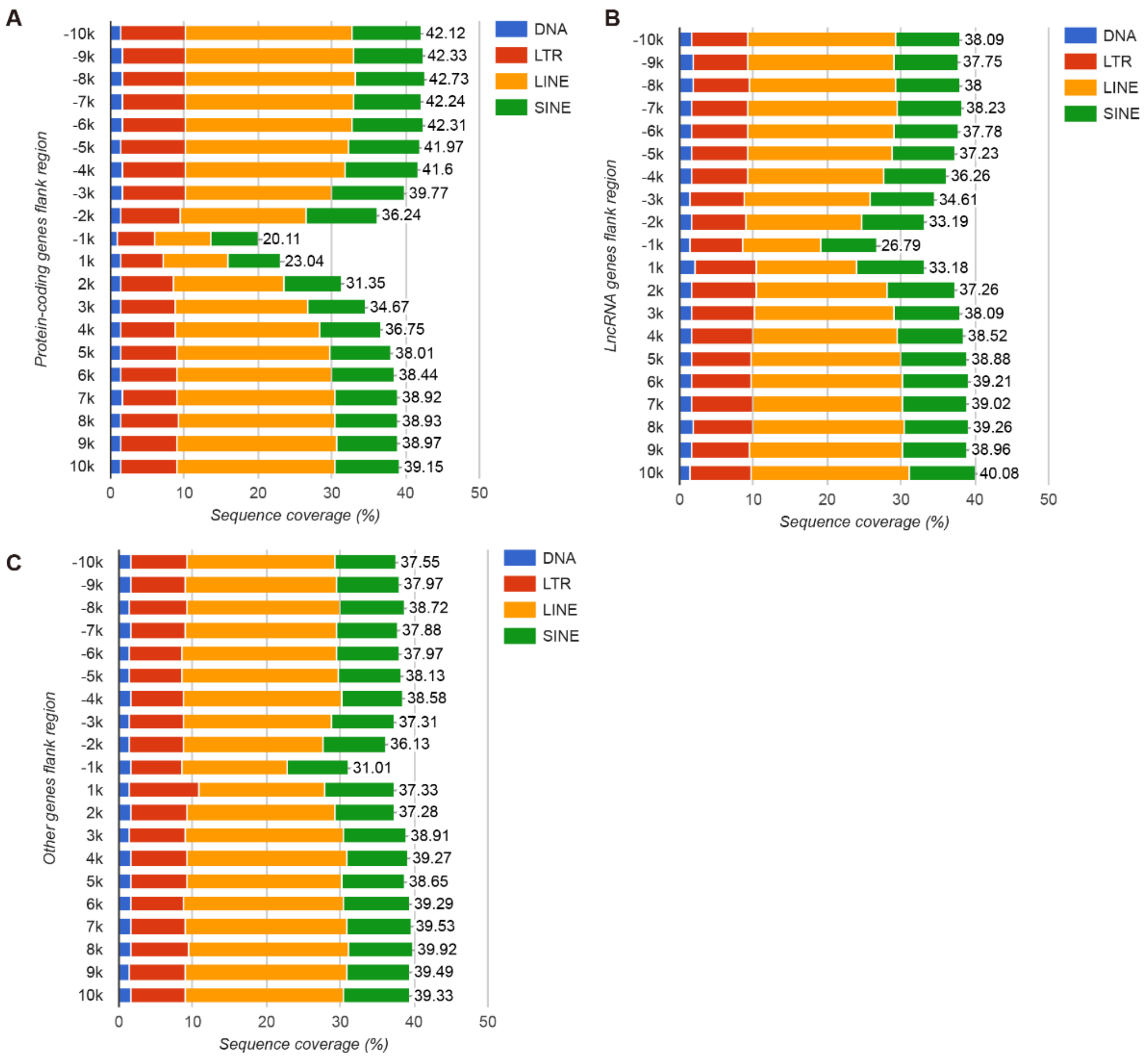

Figure S4 Density of retrotransposon elements in the flanking regions of protein-coding genes (A), lncRNA (B) and other genes (C). The ordinate axis represents the position relative to the gene, negative and positive numbers representing upstream and downstream of the gene, respectively.

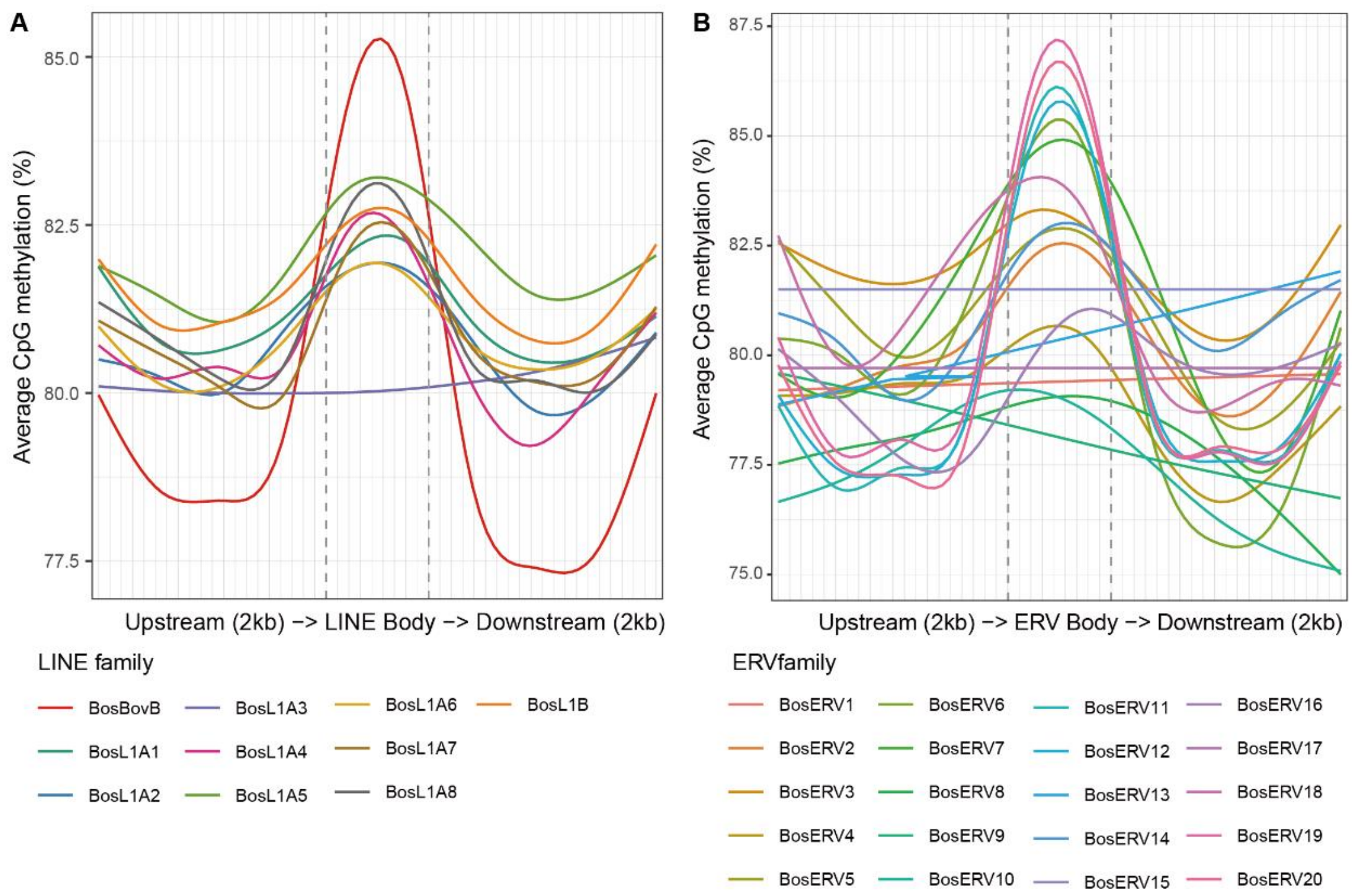

Figure S5 DNA methylation profiles across LINE (A) and ERV (B) subfamilies.

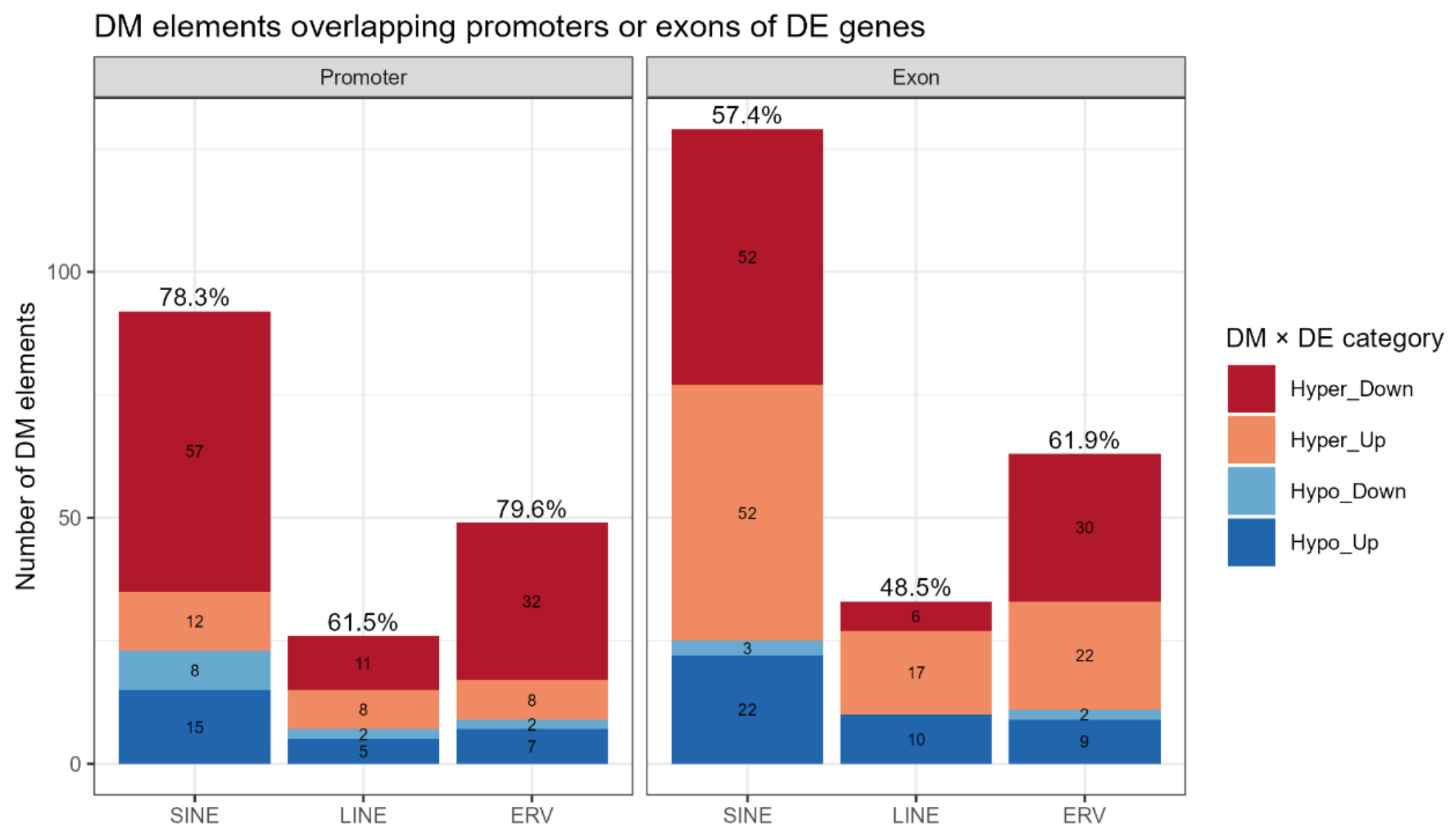

Figure S6 Number of differentially methylated (DM) retrotransposons overlapping the promoter or exons of differently expressed (DE) genes. “Hyper” and “Hypo” indicate higher or lower DNA methylation in cows with *Staphylococcus aureus*–induced subclinical mastitis (SA) compared with healthy control (CT) cows. “Up” and “Down” indicate genes that are up- or down-regulated in the SA group relative to the CT group. The number above each bar represents the percentage of DM retrotransposons showing an inverse relationship with gene expression, defined as the sum of “Hyper_Down” and “Hypo_Up”.
